# Fingerprinting the Intestinal Transport of Low-Molecular-Mass Advanced Glycation End-Products (AGEs) Using a Caco‑2 Transwell Model

**DOI:** 10.1021/acs.jafc.5c08345

**Published:** 2025-08-26

**Authors:** Xiyu Li, Sebastiaan Wesseling, Yaxin Sang, Ivonne M.C.M. Rietjens

**Affiliations:** † Division of Toxicology, 4508 Wageningen University and Research, 6708 WE Wageningen, The Netherlands; ‡ College of Food Science and Technology, 74562 Hebei Agricultural University, Baoding 071000, China

**Keywords:** Advanced glycation end products, Quantitative structure−activity relationships, Caco-2 cell transwell model, Mixture AGEs, Batch testing

## Abstract

Food-borne advanced glycation end-products could potentially contribute to the endogenous AGE accumulation within the body, albeit to a different extent for different AGEs. This study focuses on characterizing intestinal absorption and intracellular accumulation of 10 selected free low-molecular-mass (LMM) advanced glycation end-products (AGEs) to obtain insight into potential differences in their systemic bioavailability by using a Caco-2 transwell model. The findings reveal that all tested AGEs can cross the intestinal barrier through the paracellular route, albeit to a limited extent. Glycolic acid-lysine-amide (GALA) shows the highest transport percentage, reaching 1.1% ± 0.3% after 2 h, while N-ε-(carboxymethyl)­lysine (CML) displays the highest level of accumulation in intestinal cells, reaching 3.5% ± 0.6%. In contrast, cross-linked AGEs appeared to be hardly absorbed or accumulating. Passive transport likely dominates the intestinal uptake of LMM AGEs, with quantitative structure activity relationships based on maximum projection area and molar refractivity or on maximum projection area and molecular mass best describing their uptake rate. This study provides novel insights into differences in bioavailability and intracellular accumulation of LMM AGEs and the potential for fingerprinting their intestinal transport by a new approach methodology.

## Graphical abstract

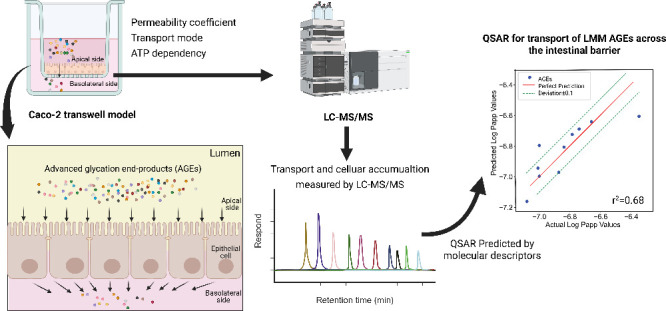

## 1 Introduction

Advanced glycation end products (AGEs) are a group of compounds formed through the reaction of sugars with amino acids. Exposure to AGEs may result from not only endogenous formation but also from external sources, such as diet.^1^ The Maillard reaction, well-known in the field of food science, has been extensively studied as a critical pathway for AGE formation.^2,3^ In the early stage of this reaction, carbonylamine condensation and molecular rearrangements occur. In the intermediate stage, rearranged products, including fructoselysine, are converted to carbonyl compounds through multiple pathways. In the late stage, reactive dialdehydes, such as glyoxal and methylglyoxal, interact with free or protein-bound amino acid nucleophilic side chains to form various AGEs.^4^ The Maillard reaction is commonly used to impart distinct flavors and attractive colors to food products. However, products formed during the Maillard reaction, including AGEs and their dialdehyde precursors, have been reported to potentially impact human health by promoting inflammation and increasing levels of reactive oxygen species (ROS),^5^ contributing to a range of adverse health effects, including metabolic dysfunction and neurological diseases, etc.^6,7^

AGEs are highly diverse and can be found in a wide range of foods, with some studies detecting over 40 different AGEs,^8^ including high-molecular-mass (HMM) AGEs, low-molecular-mass (LMM) AGEs, and their precursors. According to the classification proposed by Gerdemann et al.,^9^ LMM AGEs are defined as free or peptide-bound AGEs with a molecular mass below 12 kDa, whereas HMM AGEs refer to protein-bound forms with a molecular mass above 12 kDa. According to existing literature, LMM AGEs or their precursors may enter the systemic circulation, leading to subsequent endogenous exposure and AGE formation, whereas HMM AGEs may not be efficiently absorbed unless metabolized into LMM AGEs by host enzymes or intestinal microbiota.^10^ Therefore, this work focuses on a series of LMM AGEs shown in Figure 1. Currently, N-ε-(carboxymethyl)­lysine (CML) and N-ε-(carboxyethyl)­lysine (CEL), which result from the reaction between lysine and α-dicarbonyl compounds or from the oxidation of Amadori products, are often used as typical markers for assessing the presence of AGEs.^11,12^ Similarly, glycolic acid–lysine–amide (GALA) is another LMM AGE formed from reducing sugars through the glyoxal–imine pathway.^13^ Pyrraline, a LMM AGE formed through the reaction of lysine with reducing sugars, shows promise as a marker for dietary AGE exposure.^14^ Moreover, methylglyoxal can glycate the amino groups of arginine, producing three isomers of methylglyoxal-hydroimidazolone (MG-H), including MG-H1, and can also react with arginine to produce argpyrimidine. In addition, glyoxal and methylglyoxal can cross-link with lysine and form glyoxal-derived lysine dimer (GOLD), glyoxal-lysine-amide (GOLA), and methylglyoxal-lysine dimer (MOLD). Furthermore, arginine can also cross-link with reducing sugars forming cross-linked AGEs like pentosidine. These mentioned diverse AGEs are reported to be widely present in processed foods such as pasta, bread, and grilled meat.^15–17^

**Figure 1:**
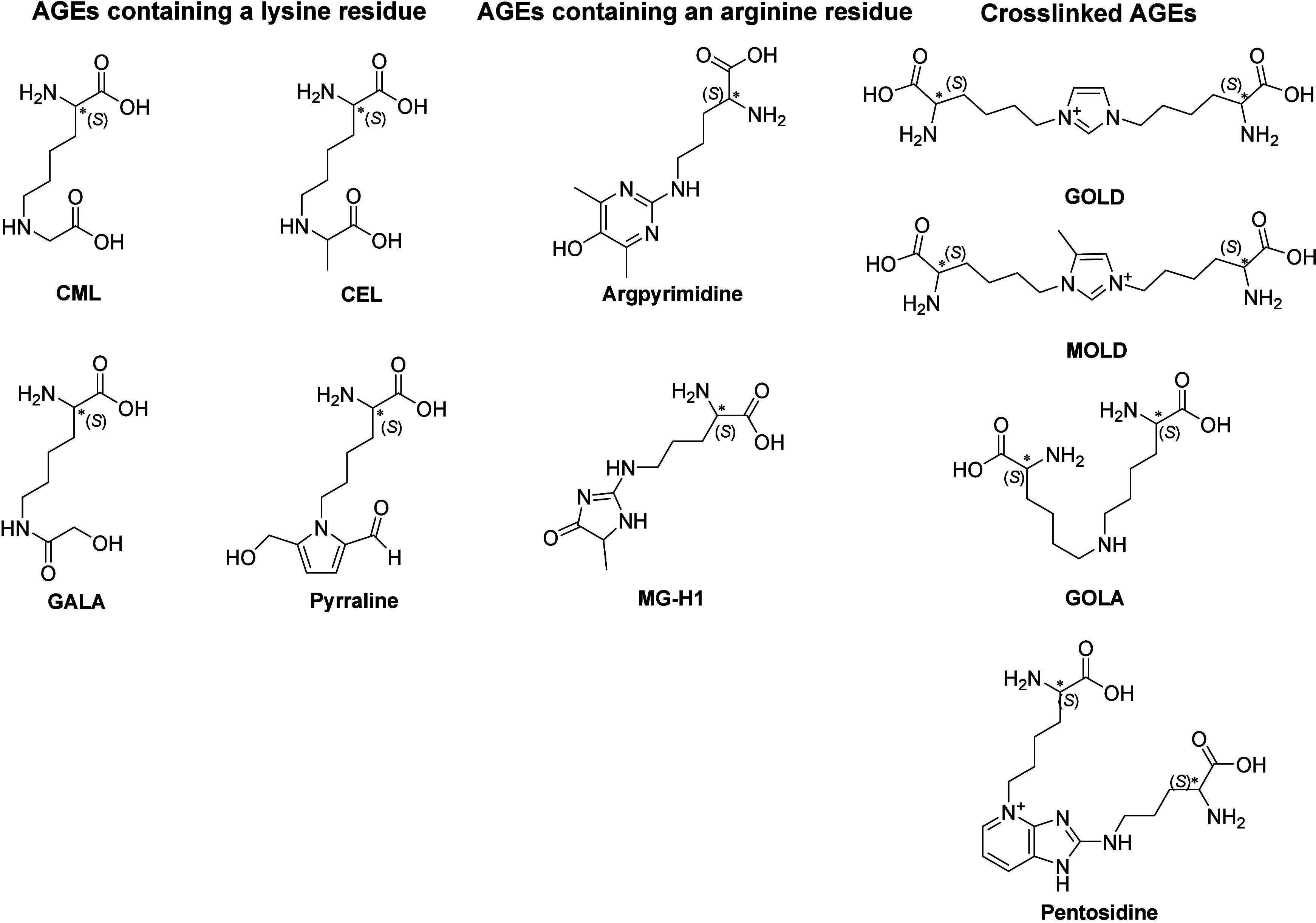
Structural formulas of the model low-molecular-mass (LMM) AGEs studied.

While some studies have investigated the bioavailability of LMM AGEs,^18,19^ our understanding of relative differences in intestinal absorption, intracellular accumulation, and transport across various LMM AGEs remains incomplete.^10^ According to previous studies, AGE precursors such as glyoxal and methylglyoxal, as well as free LMM AGEs, such as CML, most likely pass the intestinal cell barrier by passive transport,^18,20^ whereas pyrraline-containing dipeptides can also enter the intestinal cells actively via the human intestinal peptide transporter-1 (PepT1).^21^ The Caco-2 cell model has been widely used in vitro as the gold standard for studying intestinal transport,^22^ providing results that show a rank-order correlation with absorption in human subjects, and adequately describe the in vivo situation.^23^ This model also enables elucidation of potential differences in the mode of action underlying the transport of different LMM AGEs, by studying the transport characteristics in the presence of selected inhibitors or promoters.

Quantitative structure–activity relationship (QSAR) models are powerful new approach methodologies for predicting absorption based on molecular descriptors.^24^ While several QSAR frameworks and machine learning algorithms have demonstrated reliable performance in predicting transport across Caco-2 cell layers, their application to AGEs remains relatively underexplored, in part because existing AGE permeability data are scattered across different laboratories. Regarding the intestinal permeability of AGEs, the QSAR method facilitates rapid in silico screening of AGE libraries to assess their potential intestinal transport based on molecular descriptors, in some cases, even eliminating the need for subsequent in vitro or in vivo experiments.

Due to the ubiquity of the Maillard reaction in food and the heterogeneity of its products, humans are rarely exposed to dietary AGEs in isolation; instead, intestinal epithelial cells encounter highly heterogeneous mixtures of AGEs that differ in molecular size, structure, and polarity, all molecular characteristics that may influence their translocation across the intestinal barrier.^25^ It has been reported that, when multiple Maillard reaction products are administered together, they may follow different absorption and metabolic pathways in vivo.^26^ As we noted above, individual AGEs are absorbed by different routes and at different rates.^19^ Studying the transport of AGEs in mixtures rather than in isolation will generate data that better mimic actual dietary exposures. This study aims to identify potential differences in intestinal absorption, intracellular accumulation, and transport of a series of LMM AGEs using a new approach methodology (NAM) with a Caco-2 cell transwell model, which enables fingerprinting of the transport characteristics of a mixture of 10 different LMM AGEs.

## 2 Materials and Methods

### 2.1 Chemicals and Materials

N-ε-(carboxymethyl)­lysine (CML) (>96%) and N-ε-(carboxyethyl)­lysine (CEL) (>96%) were obtained from Biosynth Carbosynth (Bratislava, Slovakia). Other LMM AGEs including glycolic acid–lysine–amide (GALA) (>98%), methylglyoxal–hydroimidazolone (MG-H1) (96.6%), argpyrimidine (98.2%), glyoxal-derived lysine dimer (GOLD) (98.9%), glyoxal–lysine–amide (GOLA) (>98%), methylglyoxal–lysine dimer (MOLD) (99.8%), pentosidine (98.8%), and pyrraline (99.5%) were acquired from Iris-biotech (Marktredwitz, Germany). Dulbecco’s modified eagle medium (DMEM) with GlutaMAX, nonessential amino acids (NEAA), penicillin/streptomycin (P/S), trypsin, ethylenediaminetetraacetic acid (EDTA), 4-(2-hydroxyethyl)-1-piperazine ethanesulfonic acid (HEPES), Hanks balanced salt solution (HBSS), and phosphate buffered saline (PBS) were obtained from Gibco (Brooklyn, NY, USA). Cell Proliferation Reagent WST-1 was obtained from Roche (Mannheim, Germany). Fetal bovine serum (FBS) was obtained from Bodinco (Alkmaar, The Netherlands). Transwell 24-well plates were obtained from Corning Incorporated (Corning, NY, USA). Solvents used for liquid chromatography–mass spectrometry (LC-MS), were all high-performance liquid chromatography grade. All other reagents employed in this study were of analytical reagent grade or of superior purity.

### 2.2 Caco-2 Cell Culture

Human colon carcinoma cells (Caco-2 cells) were obtained from the American Type Culture Collection (ATCC, Rockville, MD, USA). Caco-2 cells were cultured in complete culture medium, composed of DMEM GlutaMAX with 10% FBS (v/v), 1% P/S (v/v), and 1% NEAA (v/v) at 37 °C in a 5% (v/v) CO_2_ incubator. In this study, Caco-2 cells within passages 10–15 were utilized.

### 2.3 Cytotoxicity Assay (WST-1 Assay)

The WST-1 assay was used to quantify the viability of differentiated Caco-2 cells upon exposure to the individual AGEs and their equimolar mixture. To this end, Caco-2 cells were seeded in 96-well plates at 3 × 10^4^ cells/well (100 μL) and incubated at 37 °C under 5% (v/v) CO_2_ for 20 days. The complete culture medium was refreshed on each alternate day. After full differentiation, the culture medium was removed, and the cells were washed with HBSS (containing 1% (v/v) HEPES) twice on the testing day. The same volume (100 μL) of HBSS containing a series of concentrations of the different individual test compounds or of a mixture containing all 10 AGEs was added from a 20-times-concentrated stock solution in HBSS. The test concentrations were designed based on the estimated daily intake of total AGEs (0.55–0.66 mg/(kg·day)) and high-exposure scenarios (1.4–1.8 mg/(kg day)).^27^ Assuming a worst-case scenario as LMM AGEs in which AGEs are ingested all at once and enter the gastrointestinal tract and taking into account that the volume of gastric chyme after a meal typically ranges from 0.5 to 1 L, the concentration of AGEs reaching the intestine could be as high as approximately 1.23 mmol/L. Accordingly, the concentration range for each of the 10 AGEs used in this study for the transport experiments was set at 100 μmol/L, amounting to 1 mmol/L in total, which is consistent with the potential human exposure scenarios. The cytotoxicity experiments performed on the mixture ascertained that it would not result in adverse effects on the Caco-2 cell layer. After exposure for 2 h, the absorbance at 440 and 620 nm was measured in a plate spectrophotometer (Molecular Devices, San Jose, CA, USA, Spectra Max M2). After measurement, the cell viability was calculated as

$$\mathrm{cell viability (\%)}=\frac{\left(A_{\mathrm{tested 440 nm}}-A_{\mathrm{tested 620 nm}}\right)}{\left(A_{\mathrm{control 440 nm}}-A_{\mathrm{control 620 nm}}\right)}\times 100$$

### 2.4 Establishment of the Caco-2 Cell Model and Integrity Checking

Caco-2 cells were cultured until reaching 50%–60% confluence (undifferentiated Caco-2 cells), followed by trypsinization. Then, cells were seeded at 3 × 10^4^ cells per insert in 100 μL of complete culture medium into the transwell inserts of a 24-well transwell plate, and 600 μL of complete culture medium was added to the basolateral compartments. In both the apical and basolateral compartments, the medium was renewed every other day. After 2 weeks, the transepithelial electrical resistance (TEER) value was measured using a Millicell ERS-2 Electrical Resistance System (Burlington, MA, USA) every other day. Only wells with a TEER value of >400 Ω/cm^2^ were used for transport experiments. Moreover, the TEER value was measured before and after the transport experiment to ascertain that the Caco-2 cell layer remained intact during the whole transport experiment.

### 2.5 Transport of AGEs across the Caco-2 Cell Layer

Transport experiments were performed after 20 days of differentiation of the Caco-2 cells. The culture medium was replaced with HBSS (containing 1% (v/v) HEPES) 30 min prior to testing in both the apical and basolateral compartments. After the 30 min preincubation, 100 μL of HBSS containing a mixture of the 10 different AGEs including CML, CEL, GALA, argpyrimidine, MG-H1, GOLD, GOLA, MOLD, pentosidine, and pyrraline, each at 100 μmol/L, was added to the apical compartment after removing the preincubation medium, while the basolateral medium was replaced by 600 μL of HBSS. Then, 60 μL from the basolateral side was sampled at 0, 30, 60, 90, and 120 min and supplemented with fresh 37 °C HBSS (containing 1% (v/v) HEPES). At 120 min, a 10 μL sample from the apical side was taken for calculating apical side retention (nontransported percentage). The experimental setup of Caco-2 transwell model for transport experiments is shown in Figure 2.

**Figure 2:**
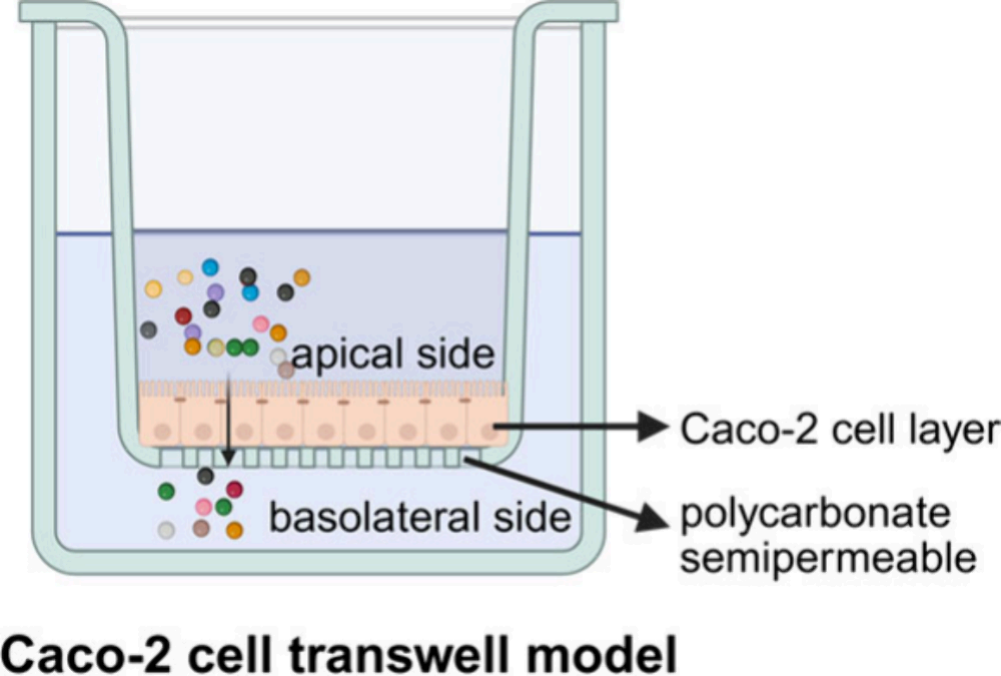
Experimental setup of the Caco-2 transwell model for transport experiments.

The apparent permeability coefficient (Papp) value was calculated according to the methodology described by Yee.^28^ In brief, the Papp value was calculated as follows:

$$\mathrm{Papp}=\frac{\Delta C\times V}{\left(A\times C_{0}\times \Delta t\right)}$$

where Δ*C* is the change in the concentration of the respective AGE at the basolateral side over the duration of the transport experiment; *V* is the volume of the basolateral compartment (cm^3^), *A* is the cell layer area (*A* = 0.33 cm^2^); *C*_0_ is the initial concentration of the respective AGE at the apical side (μmol/L); and Δ*t* is the duration of the transport experiment(s).

For further analysis of the mode of transport, transport experiments were performed in a similar way but (i) with the addition of 1 μg/mL (final concentration) cytochalasin D (which is known to promote the paracellular pathway^29^ and act on the cytoskeletal structure of Caco-2 cells^30,31^ ) to the apical compartment during the 30 min preincubation period, after which the cytochalasin D was removed, the cell layer was washed, and the transport experiment initiated; (ii) in the presence of 10 mmol/L Gly-Sar in the apical compartment during the transport experiment to competitively inhibit transport via PepT1,^31^ or (iii) at low temperature (4 °C) to limit ATP-dependent active transport.^32^

### 2.6 Accumulation of AGEs in the Caco-2 Cell Monolayer

After the transport experiment, the cell monolayer on the inset membrane was washed three times with HBSS and three times with PBS. Then, the membrane of the transwell was removed and placed in 200 μL of 65% (v/v) methanol followed by ultrasonication using Model Sonorex RK100 equipment (Bandelin, Berlin, Germany) at 80 W ultrasonic power for 30 min on ice. The samples were subsequently centrifuged at 16 000*g* for 20 min at 4 °C, and the supernatant was collected for further analysis by LC-MS.

### 2.7 LC-MS/MS Analysis of AGEs

AGEs were quantified by LC-MS using a Shimadzu Nexera XR LC-40D XR UPLC system (Kyoto, Japan) equipped with a UPLC Amide column (100 mm × 2.1 mm, 1.7 μm) without derivatization coupled with a Shimadzu LCMS-8045 triple quadrupole mass spectrometer (Kyoto, Japan). The analytical column was maintained at 40 °C throughout the quantification process. A multistep binary gradient system of water containing 0.1% (v/v) formic acid (solvent A) and acetonitrile containing 0.1% (v/v) formic acid (solvent B) was used. In the whole gradient program, the total proportion of solvents A and B is always equal to 100%. The gradient program was designed as follows: 75% solvent B to 12.5% solvent B from 0 to 10 min, followed by 12.5% solvent B from 10 to 11 min. After this, 12.5% solvent B increased to 95% solvent B from 11 to 12 min, stayed at 95% solvent B from 12 to 14 min and then decreased to the starting conditions (75% solvent B) from 14 to 14.5 min and then was kept at that level until 19 min. The initial flow rate was set to 0.30 mL/min. Subsequently, it was reduced to 0.25 mL/min at 0.3 min, which was kept until 10 min. Afterward, there was a gradual decrease, reaching 0.15 mL/min at 11 min, and this rate was maintained until 12 min. Lastly, the rate was gradually increased to 0.30 mL/min at 14 min, where it was maintained at this level until the end of the elution. Between 1 and 11 min of gradient running, the line was set to MS, and for the other time periods, the line was set to waste. The retention time and mass spectrometry parameters of individual AGEs are listed in Table S1 and the chromatograms of the samples are shown in Figure S1.

### 2.8 Quantitative Structure–Activity Relationship (QSAR) Analysis

After the transport experiments were completed, the Papp values of the 10 LMM AGEs were correlated with 17 molecular descriptors,^33^ including molecular mass, pKa1, pKa2, log *P*, the number of hydrogen-bond acceptor atoms and donor atoms, formal charge, topological polar surface area, polarizability, molar refractivity, van der Waals surface area, van der Waals volume, solvent-accessible surface area, minimum/maximum projection area, and minimum and maximum projection radius. Among these, the minimum and maximum projection area is calculated based on the van der Waals radius, and the minimum/maximum projection radius was determined from the projection conformer. Structure property prediction and calculations were performed using Calculator Plugins (Marvin 2.8.1, 2023, ChemAxon) (http://www.chemaxon.com). First, the correlation between the Papp values and each individual descriptor was determined by a linear regression analysis. To improve the prediction accuracy of the model, the Papp values were log-transformed, and a simplified regression model was constructed based on the log-transformed dataset. In a second analysis, correlations were described using two descriptors with low collinearity, which provided the best result in the single descriptor analysis. To avoid overfitting in smaller datasets, with 10 data points (or 9 data points when excluding an outlier with substantial transporter mediated translocation in addition to the passive diffusion) a maximum of two descriptors should be used for linear regression analysis. Finally, the predictive performance of the model was assessed by comparing the predicted and actual values. These steps were performed using the Python libraries Pandas,^34^ Scikit-learn,^35^ and Matplotlib.^36^

### 2.9 Statistical Analysis

All results are presented as mean ± standard error of the mean (SEM) of at least three independent transport experiments. Statistical analysis was conducted using the Statistical Package for the Social Sciences (SPSS), version 28.0.1. For cytotoxicity comparison, a one-way analysis of variance (ANOVA) was used to assess the significance of variations among different exposure concentrations, followed by Dunnett posthoc tests. For evaluation of differences between different treatment groups, independent sample *t*-tests were carried out.

## 3 Results

### 3.1 Cytotoxicity of Individual Compounds and the Mixture

As shown in Figure 3, the AGEs tested exhibited different cytotoxicities toward differentiated Caco-2 cells. CML, GOLD, and pyrraline did not affect cell viability up to the highest concentration tested. For CEL, GALA, and MOLD, there was no significant effect on cell viability at concentrations as high as 1500 μmol/L. For MG-H1, concentrations of 750 μmol/L and below did not significantly affect cell viability, while for argpyrimidine and pentosidine, concentrations of 500 μmol/L and below were not cytotoxic under the testing conditions. Caco-2 cells appeared to be most sensitive to GOLA, for which concentrations below or equal to 250 μmol/L did not affect cell viability. Results obtained for the equimolar mixture revealed that mixtures containing each AGE at 100 μmol/L or below did not affect cell viability (Figure 3k). Based on this result, an equimolar mixture containing 100 μmol/L of each of the 10 AGEs tested was used for transport experiments.

**Figure 3:**
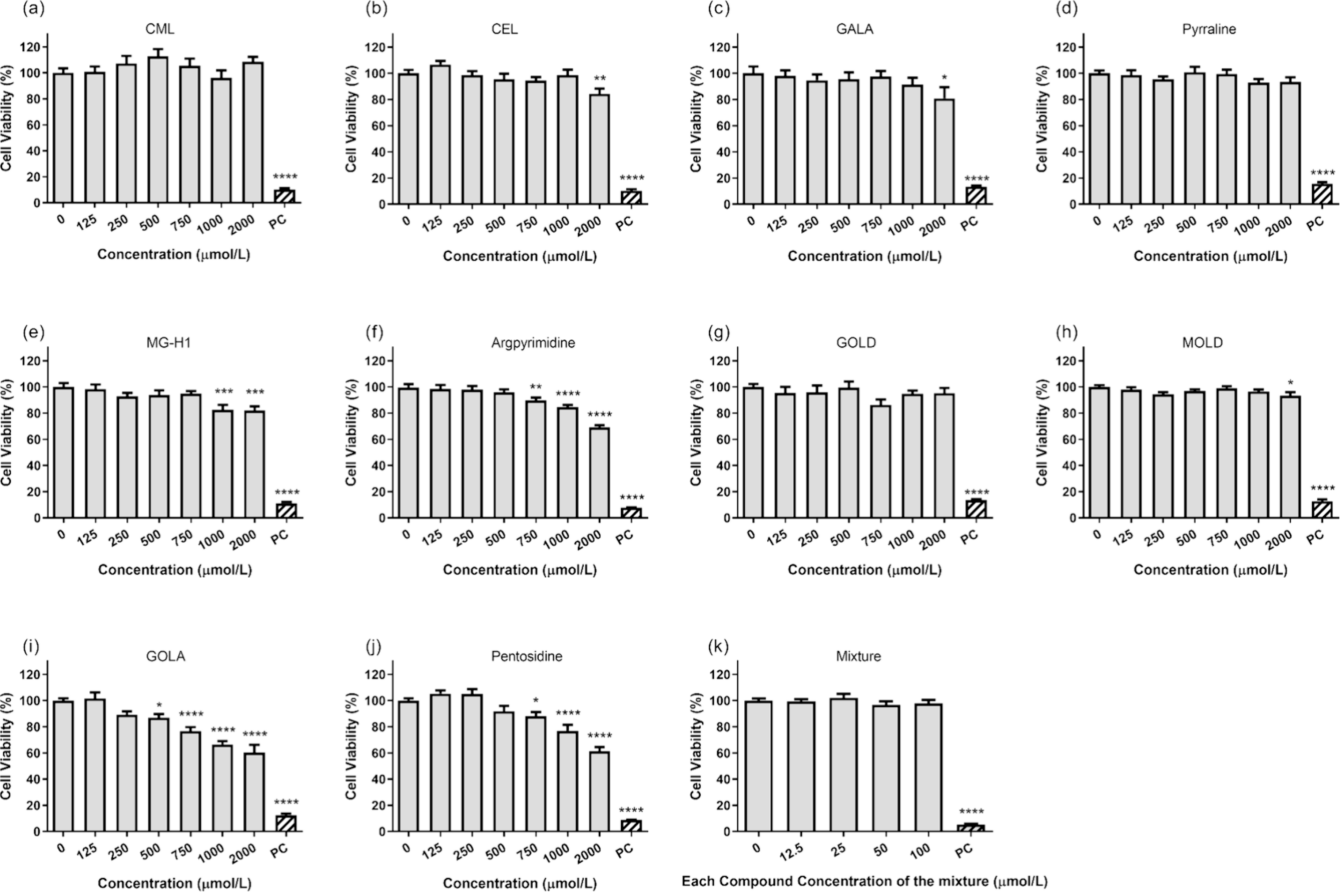
(a–j) Concentration-dependent effect of individual AGEs ((a) CML (panel (a)), CEL (panel (b)), GALA (panel (c)), pyrraline (panel (d)), MG-H1 (panel (e)), argpyrimidine (panel (f)), GOLD (panel (g)), MOLD (panel (h)), GOLA (panel (i)), pentosidine (panel (k))) and (k) their equimolar mixture, on the viability of differentiated Caco-2 cells upon 2 h of incubation. [Legend: (*) *p* ≤ 0.05, (**) *p* ≤ 0.001, (****) *p* ≤ 0.0001, based on comparisons with groups where the exposure concentration was 0 μmol/L. The positive control (PC) refers to 15 mg/L K_2_CrO_7_ in HBSS.]

### 3.2 Transport of an Equimolar Mixture of Selected AGEs across a Caco-2 Cell Layer

Figure 4 presents the results from the transport experiment in which a mixture containing equimolar concentrations of 100 μmol/L each of 10 AGEs was added to the apical side of the Caco-2 cell layer in the transwell inserts. Figure 4a presents the time-dependent transport of the different AGEs showing differences between the different AGEs, with GALA crossing the Caco-2 cell layer at a rate higher than that of the other AGEs. Figure 4b shows that the nontransported percentage of GOLA was the highest among all the tested AGEs, amounting to 96.3%, while the values of the other AGEs are around 80%–95%. Table 1 presents data regarding the total recovery of all tested AGEs, ranging from 86.2% to 96.6% of the initial amount added. Figure 4c reveals the percentage of AGEs that crossed the Caco-2 cell layer from the apical to the basolateral compartment. Consistent with the data in Figure 4a, GALA showed the highest level of transport with 1.06% of the amount added at the apical side being transported to the basolateral site after 2 h of incubation. Figure 4d presents the level of intracellular accumulation of the different AGEs in Caco-2 cells. These data reveal also substantial differences in the intracellular accumulation, with CML accumulating to the highest extent, followed by CEL and MG-H1, while for GOLD, GOLA, MOLD, and pentosidine, intracellular accumulation was lower than 0.1% or even not detectable, despite the fact that transport to the basolateral side was observed to a level amounting to 0.2 to 0.3% of the amount added at the apical side (Figure 4c). Papp values derived from the data presented in Figure 4c are shown in Table 1. Among all selected AGEs, GALA had the highest Papp value, followed by CML, MG-H1 and CEL, and the Papp value of all four cross-linked AGEs was lower than 1 × 10^–7^cm/s.

**Figure 4:**
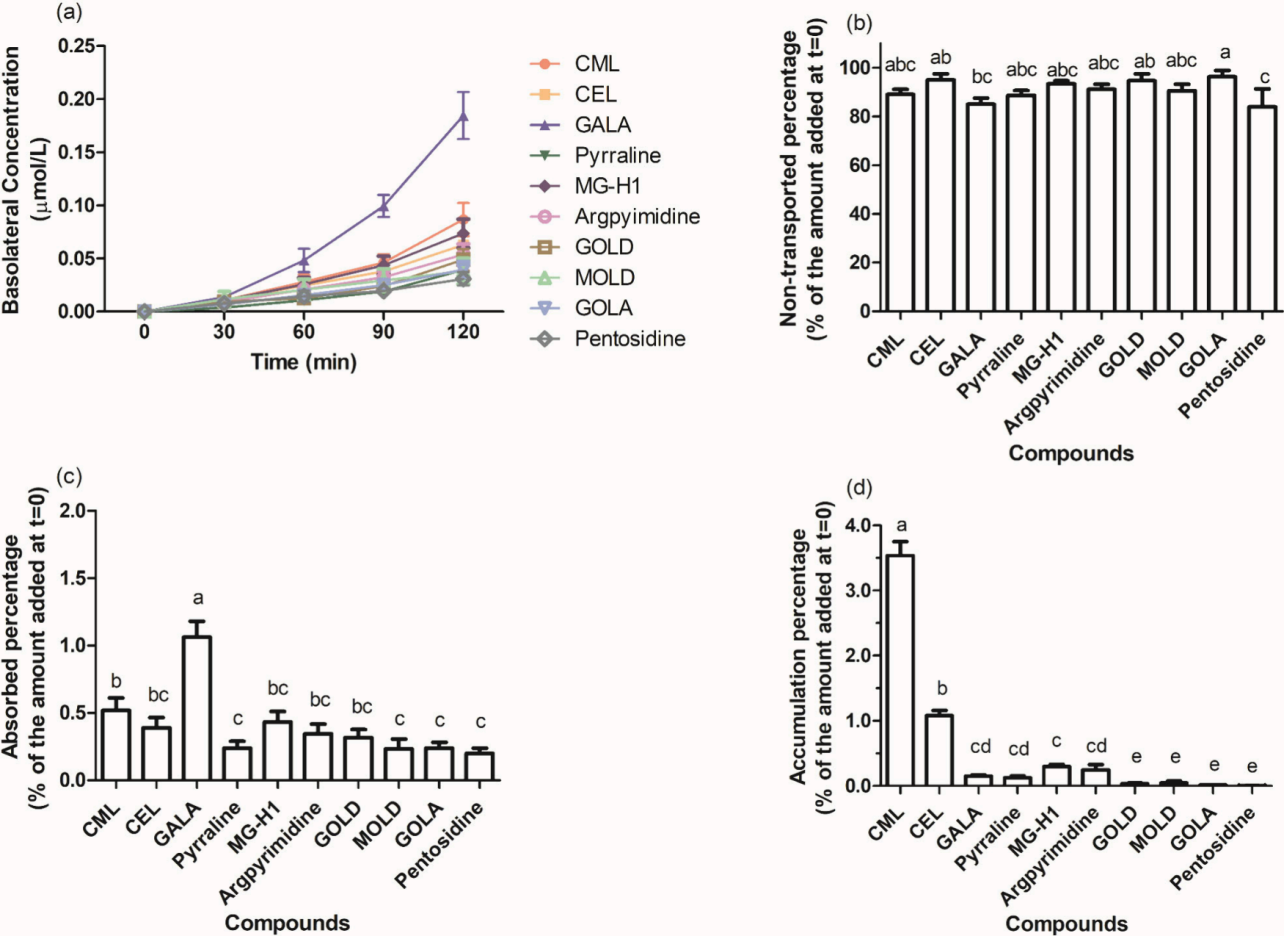
(a) Time-dependent transport of a mixture of LMM AGEs across a Caco-2 cell layer, and percentage of the amount of LMM AGEs added to the apical side that (b) was nontransported, (c) was transported to the basolateral side, and (d) accumulated inside the cells after 2 h of incubation. Notes: Bars with the same lowercase letters indicate no significant differences at *p* ≤ 0.05.

**Table 1:** Percentage of Transport and Accumulation in Caco-2 Cell Monolayer Studies with AGEs Mixture after 2 h of Transportation

			percentage (%) of the amount that was added at the apical side at *t* = 0			
compound	treatment	Papp value[Table-fn t1fn1] (× 10^–7^ cm/s)	apical side	basolateral side	accumulation	recovery
CML	standard	2.19^b^	89.0 ± 6.1 ^a^	0.5 ± 0.3 ^b^	3.5 ± 0.6 ^b^	93.1 ± 6.1 ^ab^
	cytochalasin D	5.61^a^	91.8 ± 5.7 ^a^	1.3 ± 0.6 ^a^	3.8 ± 0.9 ^b^	96.9 ± 5.8 ^a^
	Gly-Sar	2.07^b^	86.2 ± 3.6 ^a^	0.5 ± 0.1 ^b^	6.0 ± 2.1 ^a^	92.7 ± 3.1 ^ab^
	4 °C	1.05^b^	89.4 ± 5.3 ^a^	0.3 ± 0.1 ^b^	0.3 ± 0.3 ^c^	90.0 ± 5.3 ^b^
						
CEL	standard	1.64 ^b^	95.0 ± 7.00 ^a^	0.4 ± 0.2 ^a^	1.1 ± 0.2 ^a^	96.5 ± 7.2 ^a^
	cytochalasin D	5.07 ^a^	93.1 ± 4.7 ^a^	1.2 ± 0.6 ^b^	1.2 ± 0.2 ^a^	95.5 ± 5.1 ^a^
	Gly-Sar	1.49 ^b^	89.5 ± 4.8 ^a^	0.4 ± 0.1 ^a^	1.3 ± 0.2 ^a^	89.5 ± 4.8 ^a^
	4 °C	1.05 ^b^	88.8 ± 9.1 ^a^	0.3 ± 0.1 ^a^	0.0 ± 0.0 ^b^	89.1 ± 9.1 ^a^
						
GALA	standard	4.48 ^b^	85.0 ± 7.1 ^a^	1.1 ± 0.3 ^b^	0.2 ± 0.1 ^a^	86.2 ± 7.1 ^a^
	cytochalasin D	8.70 ^a^	87.4 ± 4.3 ^a^	2.1 ± 0.8 ^a^	0.2 ± 0.1 ^a^	89.7 ± 4.6 ^a^
	Gly-Sar	3.21 ^b^	84.4 ± 3.6 ^a^	0.8 ± 0.2 ^b^	0.2 ± 0.1 ^a^	85.4 ± 3.6 ^a^
	4 °C	0.95 ^c^	89.1 ± 4.5 ^a^	0.2 ± 0.1 ^c^	0.0 ± 0.0 ^b^	89.3 ± 4.5 ^a^
						
pyrraline	standard	1.00 ^b^	88.6 ± 5.5 ^a^	0.2 ± 0.1 ^b^	0.1 ± 0.1 ^a^	89.0 ± 5.6 ^a^
	cytochalasin D	3.34 ^a^	89.4 ± 4.7 ^a^	0.8 ± 0.5 ^a^	0.2 ± 0.1 ^a^	90.3 ± 4.9 ^a^
	Gly-Sar	0.80 ^b^	86.4 ± 2.8 ^a^	0.2 ± 0.1 ^b^	0.1 ± 0.0 ^a^	86.7 ± 2.8 ^a^
	4 °C	5.75 ^b^	86.9 ± 6.0 ^a^	0.1 ± 0.1 ^b^	0.0 ± 0.0 ^b^	87.1 ± 6.1 ^a^
						
MG-H1	standard	1.82 ^b^	93.4 ± 3.6 ^a^	0.4 ± 0.2 ^b^	0.3 ± 0.1 ^a^	94.1 ± 3.6 ^a^
	cytochalasin D	5.26 ^a^	93.0 ± 4.5 ^a^	1.2 ± 0.6 ^a^	0.3 ± 0.1 ^b^	94.6 ± 4.2 ^a^
	Gly-Sar	1.68 ^b^	90.1 ± 5.8 ^a^	0.4 ± 0.2 ^b^	0.3 ± 0.1 ^b^	90.8 ± 5.9 ^a^
	4 °C	1.07 ^b^	89.7 ± 5.5 ^a^	0.3 ± 0.1 ^b^	0.0 ± 0.0 ^b^	89.7 ± 5.4 ^a^
						
argpyrimidine	standard	1.45 ^b^	91.2 ± 5.9 ^ab^	0.3 ± 0.2 ^b^	0.2 ± 0.2 ^b^	91.8 ± 5.9 ^ab^
	cytochalasin D	3.95 ^a^	93.3 ± 5.2 ^a^	0.9 ± 0.6 ^a^	0.5 ± 0.3 ^a^	94.7 ± 5.4 ^a^
	Gly-Sar	1.41 ^b^	93.8 ± 5.0 ^a^	0.3 ± 0.1 ^b^	0.5 ± 0.2 ^a^	94.6 ± 5.2 ^a^
	4 °C	0.99 ^b^	87.1 ± 5.4 ^b^	0.2 ± 0.1 ^b^	0.0 ± 0.0 ^b^	87.4 ± 5.4 ^b^
						
GOLD	standard	1.34 ^b^	94.6 ± 8.1 ^ab^	0.3 ± 0.2 ^b^	0.0 ± 0.0 ^b^	95.0 ± 8.2 ^ab^
	cytochalasin D	3.20 ^a^	101.2 ± 9.4 ^a^	0.8 ± 0.3 ^a^	0.0 ± 0.0 ^ab^	102.0 ± 9.5 ^a^
	Gly-Sar	1.00 ^b^	93.7 ± 7.9 ^ab^	0.2 ± 0.1 ^b^	0.1 ± 0.0 ^a^	94.1 ± 8.0 ^ab^
	4 °C	0.69 ^b^	89.6 ± 13.2 ^b^	0.1 ± 0.1 ^b^	0.0 ± 0.0 ^c^	89.8 ± 13.2 ^b^
						
MOLD	standard	0.98 ^b^	90.6 ± 7.7 ^ab^	0.2 ± 0.2 ^b^	0.0 ± 0.1 ^a^	90.8 ± 7.9 ^ab^
	cytochalasin D	3.40 ^a^	90.8 ± 12.2 ^ab^	0.8 ± 0.6 ^a^	0.0 ± 0.1 ^a^	91.7 ± 12.6 ^ab^
	Gly-Sar	1.10 ^b^	93.5 ± 7.2 ^a^	0.3 ± 0.2 ^b^	0.0 ± 0.1 ^a^	93.8 ± 7.2 ^a^
	4 °C	0.57 ^b^	83.0 ± 6.5 ^b^	0.1 ± 0.1 ^b^	0.0 ± 0.0 ^a^	83.1 ± 6.5 ^b^
						
GOLA	standard	1.00 ^b^	96.3 ± 7.3 ^ab^	0.2 ± 0.1 ^b^	0.0 ± 0.0 ^a^	96.6 ± 7.4 ^ab^
	cytochalasin D	3.30 ^a^	101.0 ± 10.0 ^a^	0.8 ± 0.5 ^a^	0.0 ± 0.0 ^a^	101.8 ± 10.3 ^a^
	Gly-Sar	2.91 ^b^	84.0 ± 10.9 ^b^	0.7 ± 0.4 ^b^	0.0 ± 0.0 ^a^	84.7 ± 10.8 ^b^
	4 °C	0.68 ^b^	90.0 ± 11.0 ^b^	0.2 ± 0.0 ^b^	0.0 ± 0.0 ^a^	90.1 ± 10.9 ^b^
						
pentosidine	standard	0.83 ^b^	89.1 ± 12.2 ^a^	0.2 ± 0.1 ^b^	0.0 ± 0.0 ^a^	96.9 ± 12.3 ^a^
	cytochalasin D	2.78 ^a^	95.0 ± 14.2 ^a^	0.7 ± 0.4 ^a^	0.0 ± 0.0 ^a^	92.6 ± 14.5 ^a^
	Gly-Sar	0.61 ^b^	92.8 ± 10.4 ^a^	0.1 ± 0.1 ^b^	0.0 ± 0.0 ^a^	93.0 ± 10.5 ^a^
	4 °C	0.54 ^b^	83.2 ± 6.7 ^a^	0.1 ± 0.1 ^b^	0.0 ± 0.0 ^a^	83.3 ± 6.6 ^a^

* Note: Data from 120 min was used for the Papp value calculation.

### 3.3 Mode of Transport of the Different LMM AGEs

Further experiments were performed to elucidate the mode of action underlying AGE transport over the Caco-2 cells. To this end, transport studies were conducted after pretreatment of the cells with cytochalasin D, in the presence of Gly-Sar, or at 4 °C, compared to 37 °C, and the results obtained are presented in Figure 5. Cytochalasin D is a tight junction disruptor, known to promote the paracellular pathway.^29^ Pretreatment with cytochalasin D significantly increased the Papp value for all tested AGEs, by an average of +191%, indicating that all tested AGEs are able to pass the intestinal layer via the paracellular pathway (Figure 5a). The effect of cytochalasin D was most pronounced for MOLD for which pretreatment increased by over 3.5 times, compared to the Papp value in the absence of this pretreatment. The presence of Gly-Sar during the transport experiment resulted in a significant 28% reduction of the Papp value for GALA, while the Papp value of the other AGEs was unaffected (Figure 5b). Gly-Sar is a classic substrate and competitive inhibitor for PepT1 and frequently used to show a role for PepT1 in the intestinal transport of dipeptides.^31^ The addition of Gly-Sar significantly decreased the Papp value of GALA from 4.48 × 10^–7^ to 3.21 × 10^–7^, suggesting that GALA is able to be transported by PepT1 across the Caco-2 cell monolayer. Figure 5c shows the Papp values for the different AGEs obtained at 4 °C, compared to those at 37 °C, aiming to characterize the role of active transport in the translocation across the intestinal Caco-2 cell layer. The results obtained revealed that, at 4 °C,the transport of CML, GALA, and GOLD was significantly reduced. This indicates that, for CML, GALA, and GOLD, active transport contributes to their translocation across the Caco-2 cell layer. For the other 7 LMM AGEs transport was reduced by on average 37% albeit not significantly, indicating that a partial role for active transport cannot be fully excluded.^37^ Table 1 presents a full overview of the transport characteristics of the LMM AGEs under these treatments, aimed at elucidating the modes of action underlying their transport across the Caco-2 cell layer. The results in Table 1 show that the different (pre)­treatments of the Caco-2 cell layer do not significantly affect the high percentage of nontransported LMM AGE but do affect the percentage transported to the basolateral side. The data also reveal that a lack of energy during incubation at 4 °C substantially reduced the intracellular accumulation of AGEs for all compounds tested.

**Figure 5:**
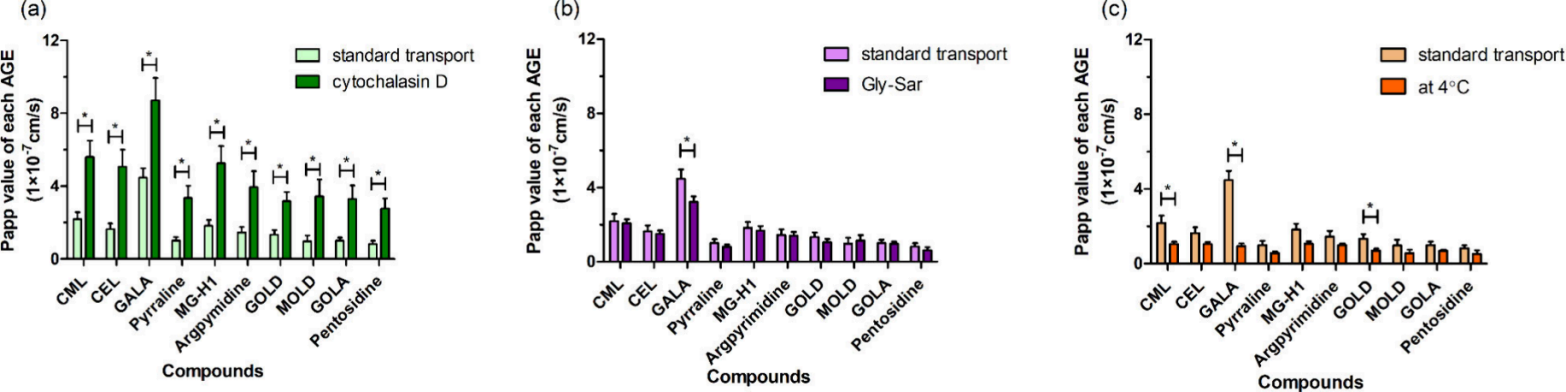
Effect of (a) cytochalasin D, (b) Gly-Sar, and (c) reduced temperature on the transport of the 10 selected low-molecular-mass (LMM) AGEs over a Caco-2 cell layer after 2 h of transportation. Note: Asterisks indicate significant differences (*p* ≤ 0.05) between the different treatments.

### 3.4 QSAR Analysis

To better understand the differences in Papp values observed for the different LMM AGE model compounds, a QSAR analysis was performed to quantify the correlation between the Papp values obtained and a series of molecular descriptors that characterize the structural and physicochemical properties of the tested AGEs. The descriptors quantified were consistent with other QSAR studies on transport characteristics and included molecular mass, pKa1, pKa2, log *P*, the number of hydrogen-bond acceptor atoms and donor atoms, formal charge, topological polar surface area, polarizability, molar refractivity, van der Waals surface area, van der Waals volume, solvent-accessible surface area, minimum and maximum projection area, and minimum and maximum projection radius.^24,38^ Table S2 and Table S3 present these molecular descriptors for the LMM AGEs. All tested AGEs showed strong hydrophilicity and zwitterionic characteristics.

All data points, gray prediction regions, and blue trend lines in each subfigure of Figure 6 present the results of the correlation analysis for the relationship between log Papp and the different descriptors. The Pearson correlation coefficients thus obtained were as follows: minimum projection radius (0.81) > molar refractivity (0.81) > polarizability (0.79) > minimum projection area (0.79) > molecular mass (0.78) > van der Waals volume (0.77) > van der Waals surface area (0.76) > maximum projection area (0.75) > solvent-accessible surface area (0.71) > topological polar surface area (0.59) > maximum projection radius (0.48) > pKa1 (0.40) > hydrogen-bond donor atoms (0.39) > formal charge (0.28) > hydrogen-bond acceptor atoms (0.27) > log *P* (0.17) > p*K*_a_ (0.02) (Figure 6). This indicates that the minimum projection radius and molar refractivity were the descriptors that best explained the variability in the log Papp values of all compounds. These descriptors are two independent descriptions of the molecules’ characteristics since the former relates to the geometry and volume of the molecule, while the latter primarily reflects to the polarity and charge distribution of the molecule (see explanation and references from ChemAxon (http://www.chemaxon.com)). Linear regression between log Papp and these two descriptors resulted in the following equation: log Papp value = −6.0525 + (−0.0887 × minimum projection radius) + (−0.0048 × molar refractivity) (*r* ^2^ = 0.68).

**Figure 6:**
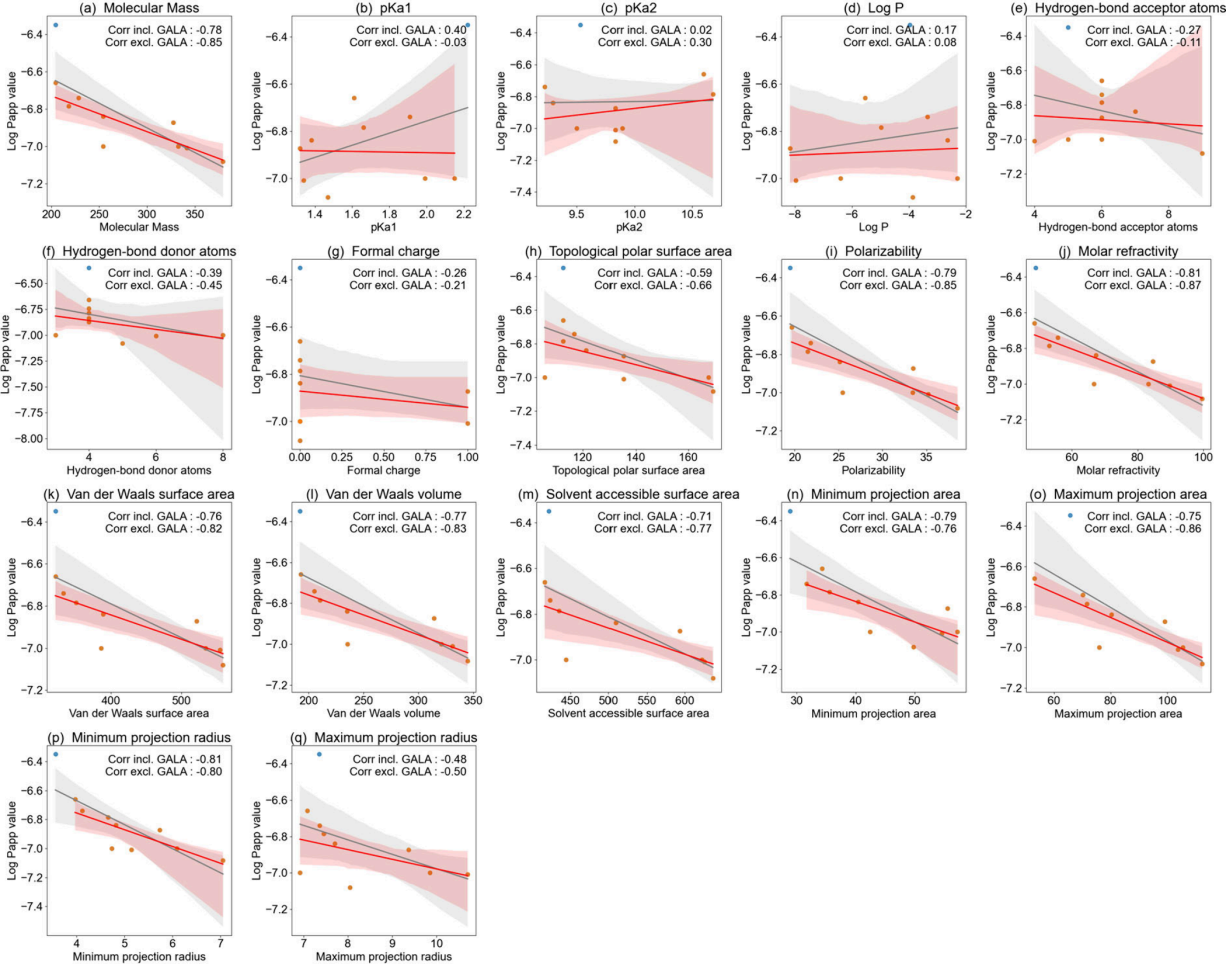
Correlation analysis of the log Papp Value with various molecular descriptors including the outlier GALA (blue data point) (in gray area and gray line) and excluding the outlier GALA (in red area and red line) for (a) molecular mass, (b) pKa1, (c) pKa2, (d) log P, (e) the number of hydrogen-bond acceptor atoms, (f) the number of hydrogen-bond donor atoms, (g) formal charge, (h) topological polar surface area, (i) polarizability, (j) molar refractivity, (k) van der Waals surface area, (l) van der Waals volume, (m) solvent-accessible surface area, (n) minimum projection area, (o) maximum projection area, (p) minimum projection radius, and (q) maximum projection radius.

The results presented in Figure 6 also reveal that especially the Papp value for GALA is an outlier in these correlations, being predicted to be lower than what is actually observed. This can be explained by the additional role of active and PepT1 mediated transport. The exclusion of this outlier from the correlation analysis is shown in Figure 5 by the red data points, red prediction regions, and red trend lines in each subfigure. Most correlation coefficients between log Papp value and the descriptor improve upon exclusion of this outlier. The ranking of the Pearson correlation coefficients changed slightly, with the results as follows: molar refractivity (0.87) > maximum projection area (0.86) > polarizability (0.85) > molecular mass (0.85) > van der Waals volume (0.83) > van der Waals surface area (0.82) > minimum projection radius (0.80) > solvent-accessible surface area (0.77) > minimum projection area (0.76) > topological polar surface area (0.66) > maximum projection radius (0.50) > hydrogen-bond donor atoms (0.45) > formal charge (0.32) > pKa2 (0.30) > hydrogen-bond acceptor atoms (0.11) > log *P* (0.08) > pKa1 (0.03). Excluding this outlier from the analysis results in the QSAR model, the molecular descriptors maximum projection area and molar refractivity best predicted the log Papp value, as expressed by the following equation: log Papp value = −6.3763+(−0.0015 × maximum projection area)+(−0.0052× molar refractivity), r^2^=0.76.

Figure 7 presents an overview of the correlation between the observed log Papp values and the log Papp values predicted by the QSAR models, both including and excluding the outlier GALA, also indicating the 10-fold deviation range (±0.1 log Papp value deviation). The results thus obtained corroborate that molecular descriptors of the LMM AGEs are important determinants of their translocation across the intestinal barrier. Including all 10 LMM AGEs (blue data points), the prediction for the log Papp value for GALA was underestimated, which is consistent with its extra mechanism for transport, in addition to the paracellular route, as shown in the studies on mode of action but not taken into account in the QSAR. The QSAR results after excluding this outlier are also presented in Figure 7a (brown data points). Almost all points fit well, within a 10-fold error range of the Papp value. The use of descriptors with high collinearity in simple QSARs models can improve the model’s predictive power and reduce the risk of overfitting.^39,40^ Therefore, the molar refractivity, molecular mass, polarizability, and van der Waals surface area are not utilized simultaneously. Since molecular mass is a crucial descriptor often playing a decisive role in transport and because it exhibits strong collinearity with the molar refractivity, an additional QSAR analysis was performed using molecular mass instead of the molar refractivity, together with maximum projection area for the QSAR analysis. The results thus obtained are shown in Figure 7b. These models fit the data equally well, with a correlation coefficient (*r* ^2^) of 0.66 for all data and a value of *r* ^2^ = 0.73 for the data excluding GALA as an outlier.

**Figure 7:**
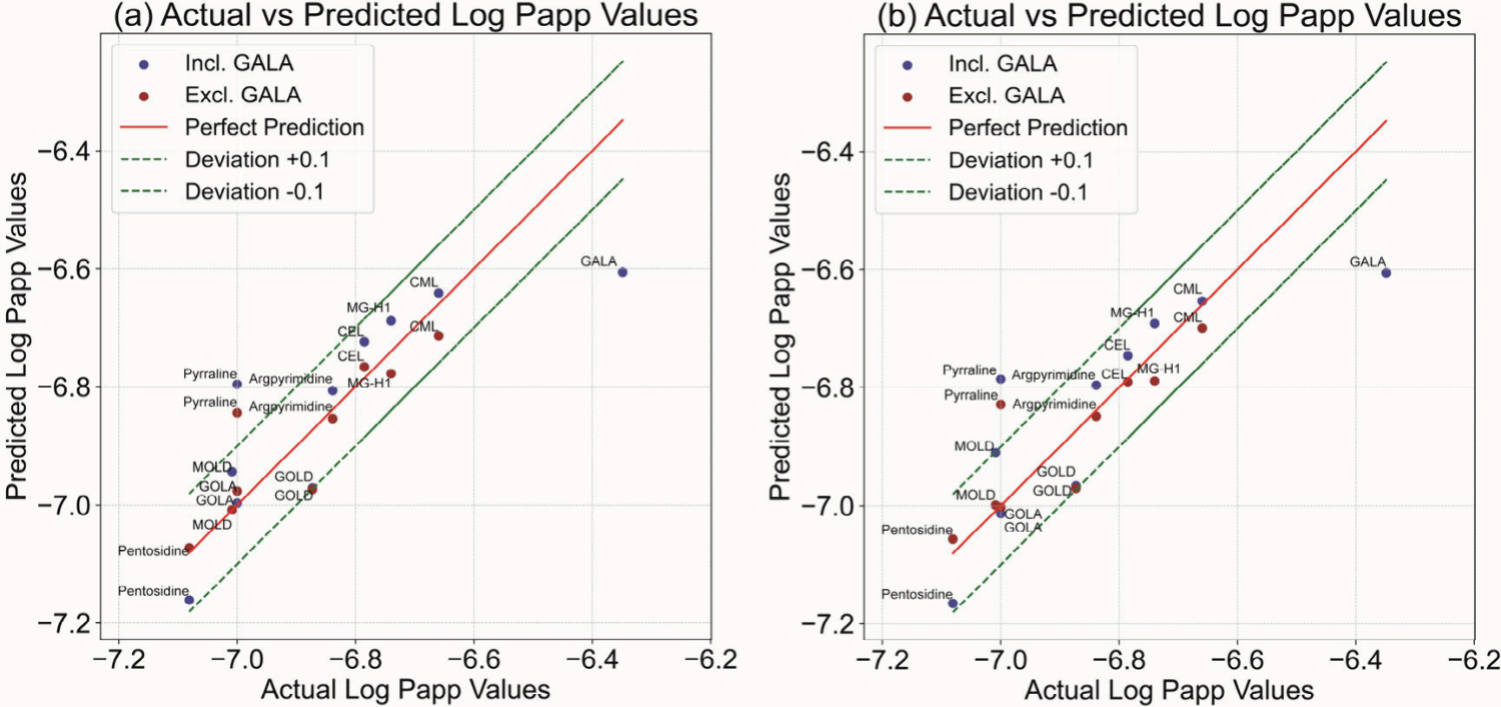
(a) Correlation between actual and predicted log Papp values using the QSAR model including the outlier GALA log Papp value = −6.0525 + (−0.0887 × minimum projection radius) + (−0.0048 × molar refractivity) (*r* ^2^ = 0.68) presented in blue points, and excluding the outlier GALA log Papp value = −6.3763 + (−0.0015 × maximum projection area) + (−0.0052 × molar refractivity) (*r* ^2^ = 0.76), presented in brown points). (b) Correlation between actual and predicted log Papp values using the QSAR model including the outlier GALA log Papp value = −6.0118 + (−0.1163 × minimum projection radius) + (−0.0009 × molecular mass) (r^2^=0.66) presented in blue points, and excluding the outlier GALA log Papp value = −6.3552 + (−0.0046 × maximum projection area) + (−0.0005 × molecular mass) (r^2^ = 0.73), presented in brown points). The red line presents the 1:1 correlation, while the green lines reflect 10-fold deviation (log Papp value = ±0.1).

## 4 Discussion

So far, dietary AGEs are recognized to be associated not only with reduced nutritional quality of the respective foods but also with adverse health effects such as food allergy, cardiovascular disease, and even cancer.^6,7^ Bioavailability is closely related to the systemic effects of dietary AGEs. Experimental evidence indicates the occurrence of intestinal absorption and systemic bioavailability of, in particular, some LMM AGEs, like especially CML,^41,42^ whereas for most other LMM AGEs, such information is absent. To enhance the understanding of the bioavailability of these other LMM AGEs, an LC-MS/MS based method was developed to simultaneously quantify 10 representative LMM AGEs and investigate their Papp values and transport mechanisms in a Caco-2 cell layer transwell transport system. The results obtained reveal that, based on the absorption standard proposed by Yee,^28^ all LMM AGEs tested are poorly absorbed, although also some remarkable differences in the transport characteristics of the different LMM AGEs were observed. GALA showed the highest percentage of transport among all LMM AGEs tested, with 1.1% of the 100 μmol/L added at the apical side being transported after 2 h, while CML is characterized by the highest level of accumulation in intestinal cells, amounting to 3.5% of the initial amount added at the apical side. Previously, the transport of CML has been quantified this LMM AGE in the Caco-2 transwell model in isolation.^20^ It is of interest to compare the Papp value obtained for CML when tested in isolation with that obtained for CML in the equimolar mixture with those of other LMM AGEs. Compared with previous findings, the Papp value of CML in the mixture was lower, amounting to 57% of that of the Papp value reported in our previous study.^20^ This difference points at competitive interactions between the different LMM AGEs affecting each other’s intestinal transport. In addition, our findings are consistent with previous reports that described relatively low absorption of free LMM AGEs, including CML, CEL, and pyrraline.^19,43^ The transport percentage of argpyrimidine and pentosidine, although relatively low (lower than 0.3%), was higher than the one reported in the literature in Caco-2 transwell experiments using the compounds in isolation at a concentration of 1 mmol/L concentration in 6-well transwell plates.^19^ Given that these AGEs can be transported through the paracellular route, it is important to recognize that differentiated Caco-2 layers typically exhibit transepithelial electrical resistance (TEER) values and tight junction integrity that exceed those of native intestinal epithelium, which may result in underestimation of passive paracellular permeability.^44^ Consequently, actual in vivo absorption of compounds could be somewhat higher than that derived from the Papp values. However, this will not affect the relative differences in uptake between the different AGEs in the same mixture. Despite these aspects, all studies point to the limited intestinal transport of these LMM AGEs, when tested in combination or in isolation. One could argue that testing a mixture better reflects the in vivo situation, where exposure to LMM AGEs via the diet may include combined exposure. The observation that Papp values from the mixture are lower than those for AGEs in isolation is likely due to competition among the compounds. And although occurring in a mixture in a dietary context, the actual concentrations of the LMM AGEs in the diet may be lower,^27^ and at these lower concentrations the competitive effects may no longer be observed. Moreover, the actual concentrations of individual AGEs may vary by several orders of magnitude, depending on the food source and dietary habits, providing another reason why such competition may be more limited under realistic dietary scenarios. However, testing the LMM AGEs at equimolar concentrations allowed a comparison of their intrinsic transport characteristics. In addition, our study shows the limited bioavailability of the LMM AGEs. HMM protein-bound AGEs are typically hydrolyzed before absorption and in vivo studies have shown that the systemic bioavailability of the respective AGEs tends to be comparable to or lower than that of the corresponding free forms of the AGEs.^42,45–47^

The results of this study also reveal differences in the intestinal absorption and transport of the various LMM AGEs. Previous reports have indicated that the absorption and transport of AGEs in the gastrointestinal tract may depend on molecular mass, charge, structure, and other molecular characteristics.^1^ In the present study, AGEs containing a lysine or an arginine residue as well as cross-linked LMM AGEs were tested and showed differences in the percentage of transport. Among them, AGEs containing a lysine or an arginine residue exhibited higher relative transport efficiency compared to cross-linked AGEs. This difference may be attributed to the larger molecular mass of cross-linked AGEs, which could affect their interaction with transporters or limit their paracellular transport due to the restricted surface area of the paracellular space or the gating function of tight junctions.^38^ Passive transcellular transport depends on both molecular size and lipophilicity, since these parameters are known to influence the diffusion coefficient through the layer. In contrast, paracellular transport has been reported to depend on both molecular size via the sieving effect and on diffusion in water.^48^

The transport mechanisms for the tested AGEs were further investigated. Previous studies indicated that pyrraline exhibits the capacity to inhibit transport of both l-lysine and Gly-Sar,^19^ suggesting that it may act as a high-affinity ligand—either as a substrate or an inhibitor in this process. This implies the presence of alternative transport mechanisms for pyrraline, in addition to passive transport reported in our study. Our results indicated PepT1 to be involved in the transport of GALA. The PepT1-mediated transport route can be affected by structure, molecular mass, hydrophobicity or peptide charges.^49^ It has been reported that, LMM AGEs may reach the systemic circulation by free diffusion, and that protein-bound AGEs will be hydrolyzed into peptide-bound AGEs, which are subsequently transported and distributed though PepT1.^1^ Although the transport of GALA was inhibited by the presence of Gly-Sar, it does not match these specific structural requirements identified for PepT1 substrates in the literature.^50^ Therefore, it remains to be established whether PepT1 is the only transporter involved in the transport of GALA.

Given that all tested LMM AGEs were able to cross the intestinal epithelial cells by passive transport and that this mode of action is known to depend on molecular descriptors, a QSAR analysis was performed to better understand the interplay between the intestinal fate of LMM AGEs and their structural characteristics. QSARs for Caco-2 cell permeability based on molecular descriptors have been previously described for other classes of chemicals.^24,33,51^ Our QSAR analysis focused on ten specific dietary LMM AGEs, with the resulting QSAR model showing a good correlation (*r* ^2^ = 0.68). The QSAR was established to evaluate and compare the intestinal absorption potential of free LMM AGEs, as their transport mode is relatively simple and can be reasonably predicted using a set of molecular descriptors with good performance. While other QSAR models have been developed to predict compound permeability for other groups of chemicals, our model offers greater specificity and comparability in the context of AGEs. Existing QSAR studies on transmembrane transport predominantly focus on passive permeability,^24^ because passive transport is primarily determined by the physicochemical properties of the molecule, such as lipophilicity, molecular size, and hydrogen-bonding capacity, and QSAR models can quantitatively relate molecular descriptors to transmembrane permeability and transport rates. The results obtained in this study revealed that minimum projection radius/area, molar refractivity, polarizability, and molecular mass appeared to correlate with the log Papp values for transmembrane permeability. Significant collinearity was observed between some of these descriptors, including molecular mass, polarizability, surface area, and molar refractivity.^48^ To define a two-descriptor-based QSAR, the descriptors identified as highly collinear were not simultaneously applied in the model. The two-descriptor-based QSAR models of the present study were based on minimum projection radius and molar refractivity or molecular mass. The maximum and minimum projection radius or area decide how easily a compound can pass through biological membranes, which can influence bioavailability and cellular behavior. Molar refractivity, which presents how much the electronic cloud around the molecule is distorted by an external electric field, reflects the molecule’s polarizability and shows a strong collinearity with molecular mass (*r* ^2^ = 0.99). Since especially GALA appeared to be transported by additional modes of action including PepT1 mediated transport, QSARs describing the primarily passive diffusion mediated transport of the LMM AGEs appeared to underestimate its translocation across the intestinal barrier. Excluding GALA from the QSAR analysis provided good correlations of the log Papp value with molar refractivity and maximum projection area (*r* ^2^ = 0.76), or with molecular mass and maximum projection area (*r* ^2^ = 0.76). Apart from the 10 AGEs examined in this study, many other dietary AGEs remain to be evaluated, in terms of their intestinal absorption profiles. To this end, the newly developed QSAR of the present study may provide a first indication about their intestinal transport. For example, for glucosepane, which is a cross-linked AGE known to be abundant in the diet, a log Papp value in the Caco-2 system was predicted by the QSAR model from Figures 7a and 7b, excluding GALA to both to be −7.1. These values indicate that the absorption of glucosepane is likely comparable to that of argpyrimidine and MG-H1 with log Papp values predicted by the same QSAR to be −7.0 and −7.2, respectively. This in silico approach may also be applied to other free AGEs not included in our experimental setup and provides a practical means to assess and compare their relative permeability. AGEs that rely mainly on carrier-mediated transport or HMM AGEs that require hydrolysis before absorption fall outside the applicability domain of this QSAR.

An interesting additional observation of the present study was the substantial difference in the intracellular accumulation of the different LMM AGEs with this accumulation being especially prominent for CML. The intracellular accumulation reached 3.5% of the initial amount added to the apical compartment after 2 h of incubation, which is comparable to the 4.5% intracellular accumulation reported in our previous study where CML underwent single testing.^20^ Consistent with this finding, cellular accumulation of CML has been reported before in an in vivo study in rats where CML was shown to accumulate in different segments of the intestine following oral dosing by gavage.^42^ Other studies have also reported that CML accumulated following long-term CML exposure by gavage or intake of a protein-bound CML-rich diet, especially in the kidneys, gut, and lungs.^42,52^ This kind of accumulation may result from the retention of hydrophilic amino acids inside the cell.^43^ At the same time, CEL, which is structurally similar to CML, exhibited considerable accumulation, albeit only at a level only about half that observed of CML. This may be related to the lower hydrophilicity of CEL compared to CML, with the more hydrophilic CML more likely to remain inside the cells.^43^ The intracellular accumulation of the tested cross-linked AGEs was less than 0.10% of the administered amount at the apical side, which indicates, together with the observation that these cross-linked AGEs were transported to a lesser extent than the other LMM AGEs, that these cross-linked AGEs are not or only hardly taken up by the intestinal cells. This difference among the different LMM AGEs is noteworthy considering that intracellular accumulation of AGEs is able to cause toxicity to cells by increasing oxidative stress, cross-linking with proteins, interacting with receptors, etc.^53^ The increasing accumulation of AGEs in cells and tissues in relation to diabetes, aging and other chronic diseases has been reported in the literature.^53,54^ It remains of interest for future studies to quantify the level of accumulation of these externally added LMM AGEs relative to the accumulation of intracellularly formed counterparts,^52,55^ and the results of the present study reveal that especially CML and CEL seem to be the model LMM AGEs of choice for such studies. Besides, the recovery rate of the AGEs, ranged between 85% and 102%. The deviation from 100% recovery can be ascribed to several factors, including analytical variability, binding of the AGEs to tissue culture transwell plates, or metabolism of the AGEs by the Caco-2 cells. In addition, losses may have occurred during the washing steps applied to ascertain that AGEs detected in the cell samples are transported into the cells and not just adhering to the cell membrane.

To conclude, a mixture of 10 selected LMM AGEs was used to study the transport of these AGEs across an intestinal cell layer using a new approach methodology. Among these AGEs, GALA exhibited the highest transport rate, and CML accumulates the most inside the cells. In contrast, cross-linked LMM AGEs show low absorption and almost no intracellular accumulation, pointing at relatively low bioavailability, compared to the other LMM AGEs. GALA can be actively transported into intestinal cells via the PepT1 transporter and passive transport, whereas CML and GOLD appear to utilize other transporters alongside passive transport. Passive transport appears to be the major driver for the intestinal uptake of the LMM AGEs, and this results in a decrease in transport with increasing minimum projection radius/area, molar refractivity, polarizability, or molecular mass. This work provides valuable knowledge on the bioavailability and cellular accumulation of different LMM AGEs and demonstrates the feasibility of using a NAM to characterize the intestinal transport of AGE mixtures.

## Supplementary Material



## Notes

Table S1, the retention time and mass spectrometry parameters of individual AGEs; Table S2, structural property coefficients of tested compounds for QSAR analysis; Table S3, geometry coefficients of tested compounds for QSAR analysis; Figure S1, extracted ion chromatographic profile of the mixture AGEs sample (PDF)

## Abbreviations Used

AGEs: advanced glycation end-products
CEL: N-ε-(carboxyethyl)­lysine
CML: N-ε-(carboxymethyl)­lysine
DMEM: Dulbecco’s modified eagle medium
EDTA: ethylenediaminetetraacetic acid
GALA: glycolic acid-lysine-amide
GOLA: glyoxal-lysine-amide
GOLD: glyoxal-derived lysine dimer
HMM: high molecular mass
HBSS: Hanks balanced salt solution
HEPES: 4-(2-hydroxyethyl)-1-piperazineethanesulfonic acid
LC-MS: liquid chromatography–mass spectrometry
LMM: low molecular mass
MG-H: methylglyoxal-hydroimidazolone
MOLD: methylglyoxal-lysine dimer
NAM: new approach methodology
NEAA: nonessential amino acids
P/S: penicillin/streptomycin
PBS: phosphate buffered saline
ROS: reactive oxygen species
TEER: trans epithelial/endothelial electrical resistance
