# Supplementary material for: Fingerprinting the Intestinal Transport of Low-Molecular-Mass Advanced Glycation End-Products (AGEs) Using a Caco‑2 Transwell Model
Source: J Agric Food Chem. 2025 Aug 26;73(36):22852–64. doi: 10.1021/acs.jafc.5c08345 (PMC12426922; doi:10.1021/acs.jafc.5c08345)
Supplement: Supplementary file 1 [file jf5c08345_si_001.pdf]

SUPPORTING INFORMATION

Fingerprinting the intestinal transport of low molecular mass advanced glycation end-products (AGEs) using a Caco-2 transwell model

Xiyu Li[1,2,*], Sebastiaan Wesseling[1], Yaxin Sang[2,*], Ivonne M.C.M. Rietjens[1]

[1] Division of Toxicology, Wageningen University and Research, 6708 WE Wageningen, The Netherlands

[2] College of Food Science and Technology, Hebei Agricultural University, Baoding 071000, China

To whom correspondence should be addressed: Xiyu Li (xiyu.li@wur.nl) and Yaxin Sang (yxsang1418@163.com)

Tables

Table S1. Retention time and mass spectrometry parameters selected for detection of individual AGEs.

Analyte	Retention time	Precursor ion $[M+H]^+$	Quantitation ion	Normalized Collision energy
CML	6.323	205.00	84.25	20
		205.00	130.25	12
CEL	5.818	219.05	84.25	20
		219.05	130.10	12
		219.05	56.15	40
GALA	3.480	205.00	142.3	14
		205.00	84.10	24
		205.00	56.15	45
Pyrraline	1.758	255.05	175.30	12
		255.05	237.15	7
		255.05	148.15	18
MG-H1	3.968	229.15	70.20	25
		229.15	114.10	15
		229.15	116.20	14
Argpyrimidine	2.866	255.05	70.25	25
		255.05	237.15	10
		255.05	70.25	16
GOLD	6.617	164.30	84.20	18
		164.30	141.30	12
		164.30	56.20	29
MOLD	6.182	171.250	84.25	17
		171.250	148.20	12
		171.250	56.20	28
GOLA	7.541	333.100	84.20	18
		333.100	141.35	10

		333.100	169.25	12
Pentosidine	6.724	190.150	135.600	13
		190.150	167.25	11
		190.150	56.20	26

Table S2 Structural property coefficients of tested compounds for QSAR analysis

Compound	Molecular Mass	AGE Type	pKa	characteristic	Log P	Hydrogen -bond acceptor atoms	Hydrogen -bond donor atoms	Formal charge	Topological polar surface area (Å^2)	Polarizability (Å^3)	Molar refractivity (cm^3/mol)	Van der Waals surface area (Å^2)
CML	204.22	AGE contains a lysine residue	1.61; 10.60	zwitterionic	-5.53	6	4	0	112.65	19.65	48.67	321.64
CEL	218.25	AGE contains a lysine residue	1.66; 10.68	zwitterionic	-4.97	6	4	0	112.65	21.47	53.16	351.06
GALA	204.22	AGE contains a lysine residue	2.22; 9.53	zwitterionic	-3.97	5	4	0	112.65	19.46	48.96	322.03
Pyrraline	254.28	AGE contains a lysine residue	1.99; 9.50	zwitterionic	-2.29	5	3	0	105.55	25.46	66.76	386.03
Argpyrimidine	254.29	AGE contains an arginine residue	1.38; 9.29	zwitterionic	-2.64	7	4	0	121.36	25.14	67.32	389.30
MG-H1	228.25	AGE contains an arginine residue	1.91; 9.22	zwitterionic	-3.35	6	4	0	116.81	21.81	55.78	333.05
GOLD	327.40	Crosslink AGE	1.32; 9.83	zwitterionic	-8.17	6	4	1	135.45	33.47	84.63	521.23
GOLA	332.40	crosslink AGE	2.15; 9.89	zwitterionic	-6.39	6	8	0	167.77	33.42	83.28	533.97
MOLD	341.40	crosslink AGE	1.34; 9.83	zwitterionic	-7.97	4	6	1	135.45	35.23	89.78	554.50
Pentosidine	379.43	crosslink AGE	1.47; 9.83	zwitterionic	-3.87	9	5	0	169.38	38.54	99.66	558.69

Table S3 Geometry coefficients of tested compounds for QSAR analysis

Compound	Van der Waals volume (Å^3)	Solvent accessible surface area (Å^2)	Minimum projection area (Å^2)	Maximum projection area (Å^2)	Minimum projection radius (Å)	Maximum projection radius (Å)
CML	192.92	415.70	34.28	53.17	3.97	7.08
CEL	210.22	434.64	35.56	71.58	4.66	7.45
GALA	192.18	420.87	28.84	65.74	3.56	7.35
Pyrraline	235.47	443.90	42.44	76.03	4.73	6.92
Argpyrimidine	234.94	508.87	40.43	80.38	4.82	7.70
MG-H1	205.19	422.86	31.66	70.14	4.12	7.36
GOLD	313.98	593.44	55.60	99.12	5.73	9.37
GOLA	320.69	622.86	57.28	105.55	6.09	9.85
MOLD	330.88	626.16	54.60	103.74	5.14	10.70
Pentosidine	344.60	637.30	49.81	112.36	7.05	8.05

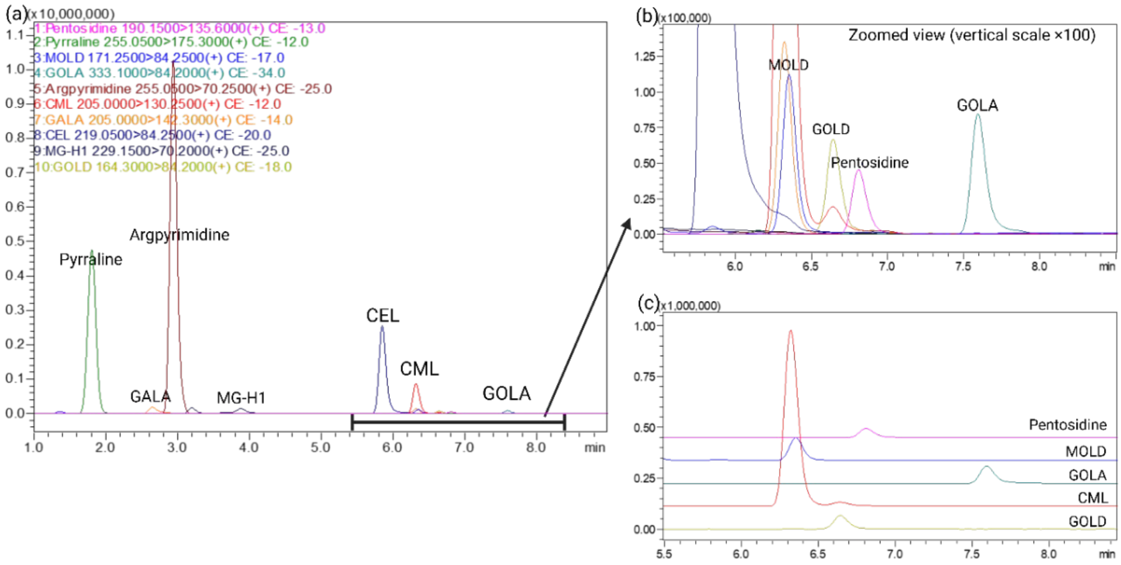

Figure S1 Extracted ion chromatographic profile of the mixture AGEs sample (apical side sample, 20-fold diluted in pH 7.4 HBSS containing 1%HEPES) showing distinct peaks corresponding to the different compounds. The different colors represent different compounds, identified based on their specific m/z fragments and retention time (Table S1). Figure A presents the whole chromatogram while figure B presents a 100-fold enlargement of part of the chromatogram and figure C presents the m/z based chromatograms used to quantify the compounds without mutual interference.
